# The effects of an intronic polymorphism in *TOMM40* and *APOE* genotypes in sporadic inclusion body myositis

**DOI:** 10.1016/j.neurobiolaging.2014.12.039

**Published:** 2015-04

**Authors:** Qiang Gang, Conceicao Bettencourt, Pedro M. Machado, Zoe Fox, Stefen Brady, Estelle Healy, Matt Parton, Janice L. Holton, David Hilton-Jones, Perry B. Shieh, Edmar Zanoteli, Boel De Paepe, Jan De Bleecker, Aziz Shaibani, Michela Ripolone, Raffaella Violano, Maurizio Moggio, Richard J. Barohn, Mazen M. Dimachkie, Marina Mora, Renato Mantegazza, Simona Zanotti, Michael G. Hanna, Henry Houlden, Muscle Study Group and the International IBM Genetics Consortium

**Affiliations:** a Department of Molecular Neuroscience, Institute of Neurology, University College London, London, UK; b Medical Research Council Centre for Neuromuscular Diseases, Institute of Neurology, University College London, London, UK; c Biomedical Research Centre, UCL and the Education Unit, Institute of Neurology, University College London, London, UK; d Nuffield Department of Clinical Neurosciences, University of Oxford, Oxford, UK; e Neuromuscular Division, Department of Neurology, University of California, Los Angeles Medical Centre, Los Angeles, CA, USA; f Department of Neurology, Medical School of the University of São Paulo (FMUSP), São Paulo, Brazil; g Department of Neurology and Neuromuscular Reference Centre, Ghent University Hospital, Ghent, Belgium; h Nerve and Muscle Centre of Texas, Houston, TX, USA; i Neuromuscular Unit, Fondazione IRCCS Ca' Granda Ospedale Maggiore Policlinico, Dino Ferrari Centre, University of Milan, Milan, Italy; j The University of Kansas Medical Centre, Kansas City, USA; k Neuromuscular Diseases and Neuroimmunology Unit, Fondazione IRCCS Isitituto Neurologico C. Besta, Milan, Italy

**Keywords:** Sporadic inclusion body myositis, sIBM, *APOE*, *TOMM40*, Age of onset

## Abstract

A previous study showed that, in carriers of the apolipoprotein E (*APOE*) genotype ε3/ε3 or ε3/ε4, the presence of a very long (VL) polyT repeat allele in “translocase of outer mitochondrial membrane 40” (*TOMM40*) was less frequent in patients with sporadic inclusion body myositis (sIBM) compared with controls and associated with a later age of sIBM symptom onset, suggesting a protective effect of this haplotype. To further investigate the influence of these genetic factors in sIBM, we analyzed a large sIBM cohort of 158 cases as part of an International sIBM Genetics Study. No significant association was found between *APOE* or *TOMM40* genotypes and the risk of developing sIBM. We found that the presence of at least 1 VL polyT repeat allele in *TOMM40* was significantly associated with about 4 years later onset of sIBM symptoms. The age of onset was delayed by 5 years when the patients were also carriers of the *APOE* genotype ε3/ε3. In addition, males were likely to have a later age of onset than females. Therefore, the *TOMM40* VL polyT repeat, although not influencing disease susceptibility, has a disease-modifying effect on sIBM, which can be enhanced by the *APOE* genotype ε3/ε3.

## 1 Introduction

Sporadic inclusion body myositis (sIBM) is known as the most common acquired myopathy among people 50 years and older (Machado et al., 2014). Given its similarities with Alzheimer's disease such as the late age of onset and the abnormal accumulation of proteins, the apolipoprotein E (*APOE*, OMIM#107741) gene has been one of the most popular genes studied in sIBM (Gang et al., 2014), but no association with sIBM disease risk was confirmed (Needham et al., 2008). Recently, the “translocase of outer mitochondrial membrane 40” (*TOMM40*, OMIM#608061) gene, which is adjacent to and in strong linkage disequilibrium with the *APOE* locus on chromosome 19, has been studied in 90 Caucasian patients with sIBM (Mastaglia et al., 2013). That study reported that among carriers of the *APOE* genotype ε3/ε3 or ε3/ε4, carriage of a very long (VL) polyT repeats in the intron 6 of *TOMM40* (rs10527454) was less frequent in sIBM compared with controls and was also associated with a later age of onset of sIBM symptoms (Mastaglia et al., 2013). To further investigate the previously reported association between *APOE*-*TOMM40* and sIBM, we genotyped *APOE* and the polyT repeat polymorphism in *TOMM40* in a large sIBM cohort of 158 affected cases from the International IBM Genetics Consortium and investigated their association with disease risk and the age of onset of sIBM.

## 2 Methods

A total of 158 DNA samples from biopsy-confirmed sIBM patients (66.5% males) were collected from 9 centres around the world. The control group comprised 127 individuals with no history of neuromuscular disease. Restriction fragment length polymorphism was used for *APOE* genotyping. Fluorescence-based fragment size analysis was performed for genotyping the polyT repeat in intron 6 of the *TOMM40* gene (rs10527454). The polyT repeat alleles were classified based on the length of repeat (*N*) as originally established by Roses et al. (2010). Genotypic frequencies for *APOE* and *TOMM40* polyT were compared between cases and controls using the chi-square or the Fisher's exact test. The associations between *APOE* genotypes, *TOMM40* polyT genotypes, ethnicity, gender, and age of onset of sIBM were analyzed by linear regression analyses. For all the analyses, *p* value <0.05 was considered statistically significant (for details, see Supplementary Data).

## 3 Results

The *APOE* ε2/ε2 genotype was absent in both the sIBM cohort and the control group. No significant differences were found between patients and controls regarding *APOE* allelic and genotypic frequencies, considering neither only Caucasian patients nor the entire cohort. The distribution of *TOMM40* polyT repeat lengths was similar in the sIBM group (range 8–36 repeats) and controls (11–33 repeats) (Supplementary Fig. 1). The frequency of neither *TOMM40* polyT repeat genotypes nor polyT alleles differed significantly between groups (Supplementary Table 1). When the analysis was performed in the combined group of carriers of ε3/ε3 and ε3/ε4 *APOE* genotypes, there was still no association between of the presence of VL polyT repeats and sIBM risk (Supplementary Table 2).

There was no significant association between different *APOE* alleles and the age of onset of sIBM. However, being a carrier of the polyT repeat, VL allele was significantly associated with a later age of onset by 3.7 years in average (95% confidence interval [CI] = 0.4, 6.9; adjusted *p* = 0.027). We repeated the analysis just for carriers of the *APOE* ε3/ε3 genotype. We found that *APOE* ε3-*TOMM40* VL allele carriers had an even later age of onset of sIBM by 4.9 years in average (95% CI = 1.1, 8.7; adjusted *p* = 0.013). Similar results were found when the analysis was restricted to Caucasians (by 4.0 years for a VL carriage, 95% CI = 0.4, 7.6; *p* = 0.028; by 5.4 years for carriage of a VL allele and *APOE* ε3/ε3 genotype, 95% CI = 1.2, 9.7; *p* = 0.013). Although not reaching statistical significance, there was also a trend for a later age of onset (by 2.7 years) in males compared with females (Table 1).

## 4 Discussion

This is the largest cohort where the influence of the *APOE* and *TOMM40* genes in sIBM disease risk and features has been investigated. Concerning *APOE*, our findings confirmed that the *APOE* ε4 allele is not a susceptibility factor for developing sIBM, which is consistent with the previous studies (Needham et al., 2008). *APOE* alleles were also not significantly associated with the age of onset of the disease. In addition, our findings did not replicate a previously reported association between *APOE*-*TOMM40* and risk of developing sIBM (Mastaglia et al., 2013). However, we observed that carriage of a VL repeat allele was significantly associated with a later age of onset of symptoms. This effect was even more pronounced among those also with the *APOE* ε3/ε3 genotype. This suggests that the *TOMM40* VL polyT repeat has a disease-modifying effect on sIBM by delaying the onset of symptoms, and the *APOE* ε3/ε3 genotype enhances this effect. Although the association between *APOE* and *TOMM40* and sIBM risk was not confirmed in our study, the finding of an association between the *TOMM40* VL polyT repeat and a later age of onset of sIBM may justify further gene expression studies in the future. In addition, there might be other variants within the *APOE*-*TOMM40* locus involved in the susceptibility to sIBM. Furthermore, identification of new genes and variants is crucial to improve our understanding of this complex disease.

## Disclosure statement

The authors have no conflicts of interest to disclose.

## Figures and Tables

**Table 1:** Influences of *APOE* alleles, *TOMM40* polyT repeat of VL length, ethnicity, and gender on the age of onset of sIBM using standard adjusted linear regression analysis

Variable	Count	Age of onset (mean ± SD), y	Adjusted analysis^a^	
			Regression coefficient (95% CI)	*p* Value^b^
Ethnicity				
Non-Caucasian	16	56.7 ± 5.7	Reference	
Caucasian	141	60.0 ± 10.0	2.8 (−2.3, 7.9)	0.28
Gender				
F	52	57.9 ± 10.4	Reference	
M	105	60.6 ± 9.3	2.7 (−0.5, 5.9)	0.095
*APOE*				
ε2/ε4^c^	6	56.8 ± 5.8		
ε3/ε3	99	60.2 ± 9.8	Reference	
ε2/ε3	19	56.9 ± 10.0	−2.9 (−7.7, 1.9)	0.23
ε3/ε4 and ε4/ε4	33	60.4 ± 9.7	1.6 (−2.4, 5.7)	0.43
*TOMM40* polyT				
No VL carriage	74	58.1 ± 9.7	Reference	
VL carriage	83	61.2 ± 9.6	3.7 (0.4, 6.9)	**0.027**
*APOE-TOMM40*				
ε3/ε3 and polyT non-VL carriage	38	57.3 ± 9.9	Reference	
ε3/ε3 and polyT VL carriage	61	62.0 ± 9.4	4.9 (1.1, 8.7)	**0.013**

Key: *APOE*, apolipoprotein E; CI, confidence interval; F, female; M, male; SD, standard deviation, sIBM, sporadic inclusion body myositis; *TOMM40*, translocase of outer mitochondrial membrane 40; VL, very long.

a Each analysis was adjusted for gender, ethnicity, tissue, and genetic factors, except for the variable under study.

b *p* value < 0.05 was considered statistically significant (marked in bold).

c ε2/ε4 was not included in the regression analysis for *APOE* alleles and the age of onset.
