# Supplementary material for: The effects of an intronic polymorphism in TOMM40 and APOE genotypes in sporadic inclusion body myositis
Source: Neurobiol Aging. 2015 Apr;36(4):1766.e1–3. doi: 10.1016/j.neurobiolaging.2014.12.039 (PMC4378665; doi:10.1016/j.neurobiolaging.2014.12.039)
Supplement: Supplementary Data [file mmc1.docx]

# Supplementary material

# 1. Introduction

Sporadic inclusion body myositis (sIBM) is known as the most common acquired myopathy among people aged over 50 years ([Machado, et al., 2014](#_ENREF_20),[Tan, et al., 2013](#_ENREF_29)). Given its similarities with Alzheimer’s disease in many features, such as the late age of onset, abnormal accumulation of proteins and mitochondrial dysfunction, focus of genetic investigation in sIBM has been laid upon known susceptibility genes for Alzheimer’s disease ([Gang, et al., 2014](#_ENREF_8)). The Apolipoprotein E (*APOE*, OMIM#107741) *ε4* allele has been confirmed as a strong risk factor for developing late-onset Alzheimer’s disease (LOAD) ([Saunders, et al., 1993](#_ENREF_28)). However, it has not been shown to be associated with sIBM disease risk ([Askanas, et al., 1996](#_ENREF_1),[Garlepp, et al., 1995](#_ENREF_9),[Gossrau, et al., 2004](#_ENREF_10),[Harrington, et al., 1995](#_ENREF_13),[Love, et al., 1996](#_ENREF_18),[Needham, et al., 2008](#_ENREF_25)), though ApoE protein deposits have been identified in both sIBM and hereditary IBM (hIBM) muscle fibres ([Askanas, et al., 1994](#_ENREF_2),[Mirabella, et al., 1996](#_ENREF_24)).

The “Translocase of Outer Mitochondrial Membrane 40” (*TOMM40*, OMIM#608061) is a gene adjacent to and in strong linkage disequilibrium with the *APOE* locus on chromosome 19 ([Yu, et al., 2007](#_ENREF_31)). It encodes the mitochondrial pore protein Tom40 involved in the transport of amyloid-β and other proteins into mitochondria ([Hansson Petersen, et al., 2008](#_ENREF_12)). After an intronic polymorphism of a variable length polyT repeat in the *TOMM40* gene (rs10527454, reported as rs10524523 in previous studies) was reported as having a significant influence on the age of onset of LOAD ([Maruszak, et al., 2012](#_ENREF_22),[Roses, et al., 2010](#_ENREF_26)), it was also recently studied in sIBM. A study on 90 Caucasian sIBM patients demonstrated that among carriers of the *APOE* genotypes ε3/ε3 or ε3/ε4, carriage of *TOMM40* very long polyT repeats was less frequent in sIBM compared to controls and was also associated with a later age of onset of sIBM symptoms ([Mastaglia, et al., 2013](#_ENREF_23)). These findings suggest that these polymorphisms may be involved in genetic susceptibility to sIBM and may have an effect on the age of onset.

To further investigate the previously reported association between *APOE*-*TOMM40* and sIBM, we genotyped *APOE* and the polyT repeat polymorphism in *TOMM40* in a large sIBM cohort of 158 affected cases from the International IBM Genetics Consortium, and investigated their association with disease risk and the age of onset of sIBM.

# 2. Materials and methods

## 2.1 Subjects

This study is part of the International IBM Genetics Consortium, a Muscle Study Group-endorsed project, which currently has members from 17 centres in seven countries around the world. For this first Consortium study, a total of 158 DNA samples from biopsy-confirmed sIBM patients (66.5% males) was collected from nine centres, including: University College London (UCL) Medical Research Council (MRC) Centre for Neuromuscular Diseases in London, United Kingdom (UK); John Radcliffe Hospital in Oxford, UK; University of California, Los Angeles (UCLA) Medical Centre in Los Angeles, United States of America (USA); Medical School of the University of São Paulo in São Paulo, Brazil; Ghent University Hospital in Ghent, Belgium; Nerve and Muscle Centre of Texas in Houston, USA; BioBank of Skeletal Muscle, Nerve Tissue, DNA and Cell Lines Neuromuscular Unit, IRCCS Foundation Ca’ Granda, in Milan, Italy; University of Kansas Medical Centre in Kansas City, USA and Neuromuscular Diseases and Neuroimmunology Unit Muscle Cell Biology Lab in Milan, Italy. The clinical information and the muscle biopsy reports of each patient were reviewed by experienced clinicians and pathologists in each centre according to the study criteria. Patients could fulfil either of the following criteria: Griggs Criteria ([Griggs, et al., 1995](#_ENREF_11),[Tawil and Griggs, 2002](#_ENREF_30)), the European Neuromuscular Centre (ENMC) 2000 Criteria ([Badrising, et al., 2000](#_ENREF_3)) or the MRC 2010 Criteria ([Hilton-Jones, et al., 2010](#_ENREF_14)). Eighty-nine DNA samples were extracted from blood, while 69 samples were extracted from muscle tissue of sIBM patients. The study was approved by the National Research Ethics Service (NRES) Committee London – Queen Square (REC reference: 12/LO/1557).

The control group comprised 127 individuals with no history of neuromuscular disease, whose DNA was extracted from pathologically confirmed normal post-mortem frontal lobe cortex or muscle tissue. All the control samples were kindly provided by the Queen Square Brain Bank in London (brain tissue) and the MRC Sudden Death Brain and Tissue Bank in Edinburgh (muscle tissue), UK.

## 2.2 Genotyping

Restriction fragment length polymorphism (RFLP) was used for *APOE* genotyping as previously described by Ingelsson et al. ([Ingelsson, et al., 2003](#_ENREF_15)).

Fluorescence-based fragment size analysis was performed for genotyping the polyT repeat in intron 6 of the *TOMM40* gene (rs10527454). Genomic DNA was amplified using the fluorescently labelled forward primer 5’FAM-TGCTGACCTCAAGCTGTCCTCTTGC-3′, and the reverse primer was 5′-GAATTGGGGAGGAAGAGAGTGGGAAA-3′ ([Cruchaga, et al., 2011](#_ENREF_6)). The PCR reactions were carried out in a total volume of 20 µl, containing 2x FastStart PCR Master (Roche), 10 pmol of forward and of reverse primers and 20 ng of DNA. The PCR conditions used were as follows: an initial denaturation at 94°C for 10 minutes; followed by 25 cycles of: 94°C for 30 seconds, 67°C for 30 seconds (-0.2°C per cycle), and 72°C for 45 seconds; 15 cycles of: 94°C for 30 seconds, 62°C for 30 seconds, and 72°C for 45 seconds; and a final extension at 72°C for 10 minutes. The expected length of the PCR product was N+247 base pairs [bp], where “N” represents the number of the polyT residues, and 247 bp was the size of polyT flanking region plus an “A” overhang at the end of the product.

The PCR product (2 µl) was mixed with 0.3 µl of Liz 500 size standard (Applied Biosystems) and 9.2 µl of HiDi formamide (Applied Biosystems) for each sample, denatured at 95°C for 3 minutes, and placed immediately on ice for 5 minutes. It was then run on an ABI3730XL Genetic Analyzer (Applied Biosystems). The genotype of each sample was determined using GeneMapper version 3.7 software (Applied Biosystems). The polyT repeat (rs10527454) alleles were classified based on the length of repeat (N) as originally established by Roses and colleagues: Short (S, ≤ 19 bp), Long (L, 20-29 bp), and Very Long (VL, ≥ 30 bp) ([Lutz, et al., 2010](#_ENREF_19),[Roses, et al., 2010](#_ENREF_26)).

## 2.3 Statistical analysis

Hardy-Weinberg Equilibrium (HWE) was tested for the distribution of *APOE* genotypes in both the sIBM patient cohort and the control group, using Arlequin Ver3.5.1.3 ([Excoffier L, 2010](#_ENREF_7)). Genotypic frequencies for *APOE* and *TOMM40* polyT were compared between cases and controls using the chi-square test or the Fisher’s exact test. The associations between *APOE* genotypes, *TOMM40* polyT genotypes, ethnicity, gender and age of onset of sIBM were analysed by linear regression analyses. Gender, tissue and ethnicity were used as covariates for the adjusted analysis in linear regression models. Stata 12 (StataCorp LP, USA) was used for the above mentioned statistical analysis. For all the analyses, *p* value < 0.05 was considered statistically significant.

# 3. Results

## 3.1 Patients demographics

## From the entire cohort of 158 sIBM patients, 142 (89.9%) were Caucasians (66.2% of which males), and 16 were from other ethnicities (68.8% of which males), including Oriental Asians, Indians and Black Africans. The age of onset was collected retrospectively. Age of onset ranged from 36 to 85 years (mean 59.7±9.7 years for the entire cohort, mean 60.0±10.0 years for Caucasians, age of onset unknown for one Caucasian patient). The mean age of the control group was 70.5±16.9 years (age unknown for one individual).

## 3.2 Association with risk of sIBM

This is the largest sIBM cohort that has been analysed so far. The *APOE* allelic and genotypic frequencies are shown in Supplementary Table 1. The *APOE* ε2/ε2 genotype was absent in both the sIBM cohort and the control group. No significant differences were found between patients and controls regarding *APOE* allelic and genotypic frequencies, neither considering only Caucasian patients nor considering the entire cohort.

Supplementary Figure 1 shows the distribution of *TOMM40* polyT repeat lengths in the sIBM group (range 8-36 repeats) and controls (range 11-33 repeats). The polyT with 13 repeats was the most common allele length among patients (41.8%) and controls (30.7%). Although patients had a higher frequency of the S allele (50.6%) compared with controls (40.6%), neither the frequency of *TOMM40* polyT repeat genotypes nor the frequency of polyT alleles differed significantly between groups (Supplementary Table 1).

To clarify the possible interaction between the *APOE* and *TOMM40* genotypes on sIBM risk, we stratified patients and controls by *APOE* genotypes (Supplementary Table 2). Within each *APOE* genotype, no significant differences were found between patients and controls. When the analysis was performed in the combined group of carriers of ε3/ε3 and ε3/ε4 *APOE* genotypes, the difference between those with and without an allele of VL polyT repeats was still not significant. Therefore, *APOE* and *TOMM40* genotypes were not associated with the risk of sIBM either independently or in combination.

## 3.3 Influence on age of onset of sIBM

When analysing the results for possible disease-modifying effects of different *APOE* alleles, because of possible opposite effects of the ε2 and ε4 alleles (by analogy with Alzheimer’s disease), six cases with the *APOE* ε2/ε4 genotype were omitted from the analysis, leaving the following three groups of cases: (a) ε2/ε3, (b) ε3/ε3, and (c) ε3/ε4 and ε4/ε4. Those with the ε2/ε3 genotype had an earlier age of onset (mean age at 56.9) compared with the other two groups (Table 1), but this difference did not reach statistical significance. However, being a carrier of the polyT repeat VL allele was significantly associated with a later age of onset by 3.7 years in average (95%CI: 0.4, 6.9; *p*=0.027, in the adjusted analysis). Since the *APOE* e3 allele with a VL allele was suggested to be associated with a later age of onset ([Mastaglia, et al., 2013](#_ENREF_23)), we repeated the analysis just for carriers of the *APOE* ε3/ε3 genotype. We found that *APOE* ε3-*TOMM40* VL allele carriers had an even later age of onset of sIBM by 4.9 years in average (95%CI: 1.1, 8.7; *p*=0.013, in the adjusted analysis). Similar results were found when the analysis was restricted to Caucasians (by 4.0 years for a VL carriage, 95%CI: 0.4, 7.6, *p*=0.028; by 5.4 years for carriage of a VL allele and *APOE* ε3/ε3 genotype, 95%CI: 1.2, 9.7, *p*=0.013). Interestingly, after adjusting for ethnicity, type of tissue and *APOE* and polyT genotypes, there was a trend for a later age of onset in males by 2.7 years when compared to females, although this did not reach statistical significance (Table 1).

# 4. Discussion

This is the largest cohort (158 patients and 127 controls) where the influence of the *APOE* and *TOMM40* genes in sIBM disease risk and features has been investigated. Concerning *APOE*, our findings confirmed that the *APOE* ε4 allele is not a susceptibility factor for developing sIBM. *APOE* alleles were also not significantly associated with the age of onset of the disease, but patients with the *ε*2 allele were likely to present slightly earlier symptoms, which is in agreement with the results from a previous study ([Needham, et al., 2008](#_ENREF_25)), where a non-significant trend towards an earlier age of onset in patients with the *ε*2 allele was also found. An increased risk of an earlier age of onset in carriers of the *APOE* *ε*2 allele has also been reported for other diseases (e.g. Machado-Joseph disease/spinocerebellar ataxia type 3, and Parkinson’s disease) ([Bettencourt, et al., 2011](#_ENREF_4),[Maraganore, et al., 2000](#_ENREF_21)). In addition, our findings did not replicate the results from a previous *APOE*-*TOMM40* study ([Mastaglia, et al., 2013](#_ENREF_23)), since in our study, carriers of the *APOE* ε3/ε3 or ε3/ε4 genotypes and a VL polyT repeat were not under-represented in sIBM compared to controls. The significant association with disease risk observed in the previous study might be a spurious effect observed in a smaller sample size population, particularly when the analysis was restricted to carriers of the *APOE* ε3/ε3 and ε3/ε4 genotypes. However, we observed that carriage of a VL repeat allele was significantly associated with a later age of onset of symptoms. This effect was even more pronounced among those also with the *APOE* ε3/ε3 genotype. This suggests that the *TOMM40* VL polyT repeat has a disease modifying effect on sIBM by delaying the onset of symptoms, and the *APOE* ε3/ε3 genotype enhances this effect.

It has been hypothesized that the polyT polymorphism may influence susceptibility to LOAD and sIBM by modulating the TOM40 protein or the APOE protein thereby altering mitochondrial function ([Mastaglia, et al., 2013](#_ENREF_23),[Roses, et al., 2010](#_ENREF_26)). Although the association between the polyT repeat locus and LOAD is still debated due to conflicting results ([Cruchaga, et al., 2011](#_ENREF_6),[Jun, et al., 2012](#_ENREF_16)), a recent expression study by Linnertz et al. showed that the VL polyT is associated with higher expression levels of *APOE* and *TOMM40* mRNA in samples from both normal and LOAD cases ([Linnertz, et al., 2014](#_ENREF_17)). Although the association between *APOE* and *TOMM40* and sIBM risk was not confirmed in our study, the finding of an association between the *TOMM40* VL polyT repeat and a later age of onset of sIBM may justify further gene expression studies in the future.

This is also the first sIBM study where muscle DNA samples from both sIBM patients and pathologically healthy controls were analysed. As sIBM is a primary muscle disease, when both blood and muscle tissue were available from same individual, muscle DNA was used for the analysis, and the regression analyses were adjusted for this potential confounder (tissue differences). Nevertheless, no significant differences in genetic data were seen between the two types of tissue (data not shown), apart from one patient with the *TOMM40* polyT genotype of L-VL in blood while the S-VL genotype was found in muscle tissue. This might be a somatic change in the polyT length caused in different tissue environments. Since the analysis focused on the influence of VL repeat length, this individual finding did not alter our findings.

Additionally, in this study males were likely to have a later age of onset than females. The age of onset in males (mean age 62, range 55 to 71 years) was very similar to females (mean age 61, range 51 to 70 years) in a previous clinical assessment study of 67 sIBM patients ([Brady, et al., 2013](#_ENREF_5)), but no statistical analysis was carried out. Further studies should be performed to clarify the disease-modifying effect of gender on sIBM.

Our study has several limitations. Due to the limited number of non-Caucasian cases, we could not conduct additional analyses stratified by ethnicity. It is therefore important to stimulate international collaboration and the recruitment of sIBM patients from different populations, in order to be able to analyse the impact of ethnicity on sIBM features. Furthermore, the data on age of onset was collected retrospectively in both this and previous studies with the potential limitation of self-reported information (recall bias). The requirements proposed by Roses and his colleagues for future replication studies of the *APOE*-*TOMM40* association with the age of onset of AD ([Roses, et al., 2013](#_ENREF_27)) should also be considered in future sIBM studies.

In summary, a sIBM disease modifying effect of the *TOMM40* VL polyT repeat was found in this study, and this effect was further enhanced by the *APOE* ε3/ε3 genotype. This was associated with a later sIBM symptom onset. Although this polyT polymorphism is not associated with the risk of sIBM, there might be other variants within the *APOE*-*TOMM40* loci involved in the susceptibility to sIBM. In addition, various other genes, including genes related to immune, degenerative and mitochondrial pathways may contribute to sIBM disease susceptibility ([Gang, et al., 2014](#_ENREF_8)). Identification of new genes and variants is crucial to improve our understanding of this complex disease.

**Authors’ contributions:** Q.G. contributed to all the experimental work, data analysis and drafting the first version of manuscript. C.B. and Z.F. contributed to the statistical plan. C.B and P.M. also contributed to drafting the first version of the manuscript. All authors contributed to sample collection and to the critical revision process and approved the final manuscript. M.G.H, H.H., and P.M. are principal investigators of the International IBM Consortium Genetics Study.

**Acknowledgements:** Q.G. is supported by a University College London (UCL) Impact Studentship and the Chinese Scholarship Council. We are grateful to the Medical Research Council (MRC) and The Wellcome Trust for the support to this study. P.M.M. is funded by a Post-Doctoral Research Fellowship award from the National Institute for Health Research (NIHR). M.G.H. is supported by a MRC Centre grant 2013-2018 (MR/K000608/01). The International IBM Consortium Genetic Study is also supported by an MRC grant (MR/J004758/1). We thank all the study collaborators from UK, USA, Australia and Europe, and especially thank all the patients and their families for participating in our study. This publication was supported by researchers at the NIHR University College London Hospitals Biomedical Research Centre. The views expressed are those of the author and not necessarily those of the National Health Service (NHS), the NIHR or the Department of Health. We wish to acknowledge the Queen Square Brain Bank in London and the MRC Sudden Death Brain and Tissue Bank in Edinburgh, UK, which provided us the control tissues. We also wish to acknowledge “The BioBank of Skeletal Muscle, Nerve Tissue, DNA and Cell Lines” member of the Telethon Network of Genetic Biobanks (grant number GTB12001), funded by Telethon Italy, and the EuroBioBank network, provided us with case specimens.

**We also acknowledge all the participants of the International IBM Genetics Consortium and the Muscle Study Group, who have endorsed this project:**

*#List of participants:*

Pedro M. Machado, Henry Houlden, Michael G. Hanna, Qiang Gang, Conceicao Bettencourt, Estelle Healy, Matt Parton, and Janice L. Holton; Department of Molecular Neuroscience, and MRC Centre for Neuromuscular Diseases, Institute of Neurology, University College London, Queen Square, London, UK.

Stefen Brady and David Hilton-Jones; Nuffield Department of Clinical Neurosciences, University of Oxford, UK.

Perry B. Shieh; Neuromuscular Division, Department of Neurology, UCLA Medical Centre, USA.

Edmar Zanoteli; Department of Neurology, Medical School of the University of São Paulo (FMUSP), São Paul, Brazil.

Boel De Paepe and Jan De Bleecker; Ghent University Hospital, Department of Neurology & Neuromuscular Reference Centre, Ghent, Belgium.

Aziz Shaibani; Nerve and Muscle Centre of Texas, Houston, Texas, USA.

Michela Ripolone, Raffaella Violano, and Maurizio Moggio; Neuromuscular Unit, IRCCS Foundation Ca’ Granda Ospedale Maggiore Policlinico, Dino Ferrari Centre, University of Milan, Milan, Italy.

Richard J. Barohn, Mazen M. Dimachkie, April L. McVey, Mamatha Pasnoor, Melanie Glenn, Omar Jawdat, Jeffrey Statland, and Gabrielle Rico; The University of Kansas Medical Centre, Kansas City, USA.

Marina Mora, Renato Mantegazza, and Simona Zanotti; Neuromuscular Diseases and Neuroimmunology Unit, Fondazione IRCCS Isitituto Neurologico C. Besta, Milano, Italy.

Merrilee Needham and Frank Mastaglia; Western Australian Neurosciences Research Institute (WANRI), University of Western Australia and Murdoch University, Fiona Stanley Hospital, Perth, Australia.

Christina Liang; Royal North Shore Hospital, New South Wales, Australia.

Marinos C. Dalakas and Angie Biba; Neuroimmunology Unit , Department of Pathophysiology, National University of Athens Medical School, Athens, Greece.

Hector Chinoy and James B. Lilleker; Centre for Musculoskeletal Research, Institute of Inflammation and Repair, Faculty of Medical and Human Sciences, University of Manchester, Manchester, UK.

Janine Lamb and Hazel Platt; Centre for Integrated Genomic Medical Research, Institute of Population Health, Faculty of Medical and Human Sciences, University of Manchester, Manchester, UK

Robert G. Cooper; MRC/ARUK Institute for Ageing and Chronic Disease, University of Liverpool, Liverpool, UK.

James A.L. Miller; Department of Neurology, Newcastle Upon Tyne Hospitals NHS Trust, UK.

Mark Roberts; Salford Royal NHS Foundation Trust, UK.

Elizabeth Househam, David Hilton, and Aditya Shivane; Derriford Hospital, Plymouth, UK.

Amy Bartlett and John T. Kissel; The Ohio State University Wexner Medical Center, USA.

Heidi Runk and Matthew Wicklund; Penn State Milton S. Hershey Medical Center, USA.

**Declaration of interests:** All authors have no competing interests.

**References**

Askanas, V., Engel, W.K., Mirabella, M., Weisgraber, K.H., Saunders, A.M., Roses, A.D., McFerrin, J. 1996. Apolipoprotein E alleles in sporadic inclusion-body myositis and hereditary inclusion-body myopathy. Annals of neurology 40(2), 264-5. doi:10.1002/ana.410400223.

Askanas, V., Mirabella, M., Engel, W.K., Alvarez, R.B., Weisgraber, K.H. 1994. Apolipoprotein E immunoreactive deposits in inclusion-body muscle diseases. Lancet 343(8893), 364-5.

Badrising, U.A., Maat-Schieman, M., van Duinen, S.G., Breedveld, F., van Doorn, P., van Engelen, B., van den Hoogen, F., Hoogendijk, J., Howeler, C., de Jager, A., Jennekens, F., Koehler, P., van der Leeuw, H., de Visser, M., Verschuuren, J.J., Wintzen, A.R. 2000. Epidemiology of inclusion body myositis in the Netherlands: a nationwide study. Neurology 55(9), 1385-7.

Bettencourt, C., Raposo, M., Kazachkova, N., Cymbron, T., Santos, C., Kay, T., Vasconcelos, J., Maciel, P., Donis, K.C., Saraiva-Pereira, M.L., Jardim, L.B., Sequeiros, J., Lima, M. 2011. The APOE epsilon2 allele increases the risk of earlier age of onset in Machado-Joseph disease. Archives of neurology 68(12), 1580-3. doi:10.1001/archneurol.2011.636.

Brady, S., Squier, W., Hilton-Jones, D. 2013. Clinical assessment determines the diagnosis of inclusion body myositis independently of pathological features. Journal of neurology, neurosurgery, and psychiatry 84(11), 1240-6. doi:10.1136/jnnp-2013-305690.

Cruchaga, C., Nowotny, P., Kauwe, J.S., Ridge, P.G., Mayo, K., Bertelsen, S., Hinrichs, A., Fagan, A.M., Holtzman, D.M., Morris, J.C., Goate, A.M. 2011. Association and expression analyses with single-nucleotide polymorphisms in TOMM40 in Alzheimer disease. Archives of neurology 68(8), 1013-9. doi:10.1001/archneurol.2011.155.

Excoffier L, L.H. 2010. Arlequin suite ver 3.5: A new series of programs to perform population genetics analyses under Linux and Windows. Molecular Ecology Resources (10).

Gang, Q., Bettencourt, C., Machado, P., Hanna, M.G., Houlden, H. 2014. Sporadic inclusion body myositis: the genetic contributions to the pathogenesis. Orphanet journal of rare diseases 9(1), 88. doi:10.1186/1750-1172-9-88.

Garlepp, M.J., Tabarias, H., van Bockxmeer, F.M., Zilko, P.J., Laing, B., Mastaglia, F.L. 1995. Apolipoprotein E epsilon 4 in inclusion body myositis. Annals of neurology 38(6), 957-9. doi:10.1002/ana.410380619.

Gossrau, G., Gestrich, B., Koch, R., Wunderlich, C., Schroder, J.M., Schroeder, S., Reichmann, H., Lampe, J.B. 2004. Apolipoprotein E and alpha-1-antichymotrypsin polymorphisms in sporadic inclusion body myositis. European neurology 51(4), 215-20. doi:10.1159/000078488.

Griggs, R.C., Askanas, V., DiMauro, S., Engel, A., Karpati, G., Mendell, J.R., Rowland, L.P. 1995. Inclusion body myositis and myopathies. Annals of neurology 38(5), 705-13. doi:10.1002/ana.410380504.

Hansson Petersen, C.A., Alikhani, N., Behbahani, H., Wiehager, B., Pavlov, P.F., Alafuzoff, I., Leinonen, V., Ito, A., Winblad, B., Glaser, E., Ankarcrona, M. 2008. The amyloid beta-peptide is imported into mitochondria via the TOM import machinery and localized to mitochondrial cristae. Proceedings of the National Academy of Sciences of the United States of America 105(35), 13145-50. doi:10.1073/pnas.0806192105.

Harrington, C.R., Anderson, J.R., Chan, K.K. 1995. Apolipoprotein E type epsilon 4 allele frequency is not increased in patients with sporadic inclusion-body myositis. Neuroscience letters 183(1-2), 35-8.

Hilton-Jones, D., Miller, A., Parton, M., Holton, J., Sewry, C., Hanna, M.G. 2010. Inclusion body myositis: MRC Centre for Neuromuscular Diseases, IBM workshop, London, 13 June 2008. Neuromuscular disorders : NMD 20(2), 142-7. doi:10.1016/j.nmd.2009.11.003.

Ingelsson, M., Shin, Y., Irizarry, M.C., Hyman, B.T., Lilius, L., Forsell, C., Graff, C. 2003. Genotyping of apolipoprotein E: comparative evaluation of different protocols. Current protocols in human genetics / editorial board, Jonathan L Haines [et al] Chapter 9, Unit9 14. doi:10.1002/0471142905.hg0914s38.

Jun, G., Vardarajan, B.N., Buros, J., Yu, C.E., Hawk, M.V., Dombroski, B.A., Crane, P.K., Larson, E.B., Mayeux, R., Haines, J.L., Lunetta, K.L., Pericak-Vance, M.A., Schellenberg, G.D., Farrer, L.A. 2012. Comprehensive search for Alzheimer disease susceptibility loci in the APOE region. Archives of neurology 69(10), 1270-9. doi:10.1001/archneurol.2012.2052.

Linnertz, C., Anderson, L., Gottschalk, W., Crenshaw, D., Lutz, M.W., Allen, J., Saith, S., Mihovilovic, M., Burke, J.R., Welsh-Bohmer, K.A., Roses, A.D., Chiba-Falek, O. 2014. The cis-regulatory effect of an Alzheimer's disease-associated poly-T locus on expression of TOMM40 and apolipoprotein E genes. Alzheimer's & dementia : the journal of the Alzheimer's Association. doi:10.1016/j.jalz.2013.08.280.

Love, S., Nicoll, J.A., Lowe, J., Sherriff, F. 1996. Apolipoprotein E allele frequencies in sporadic inclusion body myositis. Muscle & nerve 19(12), 1605-7. doi:10.1002/(SICI)1097-4598(199612)19:12&lt;1605::AID-MUS11&gt;3.0.CO;2-Q.

Lutz, M.W., Crenshaw, D.G., Saunders, A.M., Roses, A.D. 2010. Genetic variation at a single locus and age of onset for Alzheimer's disease. Alzheimer's & dementia : the journal of the Alzheimer's Association 6(2), 125-31. doi:10.1016/j.jalz.2010.01.011.

Machado, P.M., Dimachkie, M.M., Barohn, R.J. 2014. Sporadic inclusion body myositis: new insights and potential therapy. Current opinion in neurology 27(5), 591-8. doi:10.1097/wco.0000000000000129.

Maraganore, D.M., Farrer, M.J., Hardy, J.A., McDonnell, S.K., Schaid, D.J., Rocca, W.A. 2000. Case-control study of debrisoquine 4-hydroxylase, N-acetyltransferase 2, and apolipoprotein E gene polymorphisms in Parkinson's disease. Movement disorders : official journal of the Movement Disorder Society 15(4), 714-9.

Maruszak, A., Peplonska, B., Safranow, K., Chodakowska-Zebrowska, M., Barcikowska, M., Zekanowski, C. 2012. TOMM40 rs10524523 polymorphism's role in late-onset Alzheimer's disease and in longevity. Journal of Alzheimer's disease : JAD 28(2), 309-22. doi:10.3233/jad-2011-110743.

Mastaglia, F.L., Rojana-Udomsart, A., James, I., Needham, M., Day, T.J., Kiers, L., Corbett, J.A., Saunders, A.M., Lutz, M.W., Roses, A.D. 2013. Polymorphism in the TOMM40 gene modifies the risk of developing sporadic inclusion body myositis and the age of onset of symptoms. Neuromuscular disorders : NMD. doi:10.1016/j.nmd.2013.09.008.

Mirabella, M., Alvarez, R.B., Engel, W.K., Weisgraber, K.H., Askanas, V. 1996. Apolipoprotein E and apolipoprotein E messenger RNA in muscle of inclusion body myositis and myopathies. Annals of neurology 40(6), 864-72. doi:10.1002/ana.410400608.

Needham, M., Hooper, A., James, I., van Bockxmeer, F., Corbett, A., Day, T., Garlepp, M.J., Mastaglia, F.L. 2008. Apolipoprotein epsilon alleles in sporadic inclusion body myositis: a reappraisal. Neuromuscular disorders : NMD 18(2), 150-2. doi:10.1016/j.nmd.2007.09.005.

Roses, A.D., Lutz, M.W., Amrine-Madsen, H., Saunders, A.M., Crenshaw, D.G., Sundseth, S.S., Huentelman, M.J., Welsh-Bohmer, K.A., Reiman, E.M. 2010. A TOMM40 variable-length polymorphism predicts the age of late-onset Alzheimer's disease. The pharmacogenomics journal 10(5), 375-84. doi:10.1038/tpj.2009.69.

Roses, A.D., Lutz, M.W., Crenshaw, D.G., Grossman, I., Saunders, A.M., Gottschalk, W.K. 2013. TOMM40 and APOE: Requirements for replication studies of association with age of disease onset and enrichment of a clinical trial. Alzheimer's & dementia : the journal of the Alzheimer's Association 9(2), 132-6. doi:10.1016/j.jalz.2012.10.009.

Saunders, A.M., Strittmatter, W.J., Schmechel, D., George-Hyslop, P.H., Pericak-Vance, M.A., Joo, S.H., Rosi, B.L., Gusella, J.F., Crapper-MacLachlan, D.R., Alberts, M.J., et al. 1993. Association of apolipoprotein E allele epsilon 4 with late-onset familial and sporadic Alzheimer's disease. Neurology 43(8), 1467-72.

Tan, J.A., Roberts-Thomson, P.J., Blumbergs, P., Hakendorf, P., Cox, S.R., Limaye, V. 2013. Incidence and prevalence of idiopathic inflammatory myopathies in South Australia: a 30-year epidemiologic study of histology-proven cases. International journal of rheumatic diseases 16(3), 331-8. doi:10.1111/j.1756-185X.2011.01669.x.

Tawil, R., Griggs, R.C. 2002. Inclusion body myositis. Current opinion in rheumatology 14(6), 653-7.

Yu, C.E., Seltman, H., Peskind, E.R., Galloway, N., Zhou, P.X., Rosenthal, E., Wijsman, E.M., Tsuang, D.W., Devlin, B., Schellenberg, G.D. 2007. Comprehensive analysis of APOE and selected proximate markers for late-onset Alzheimer's disease: patterns of linkage disequilibrium and disease/marker association. Genomics 89(6), 655-65. doi:10.1016/j.ygeno.2007.02.002.

**Supplementary Table 1. Frequencies of *APOE* and *TOMM40* polyT repeat genotypes and alleles in sIBM patients and controls.**

|  | sIBM Patients  N=142 Caucasians \| N= 16 Other | | Controls  N=127 | | Total  N=285 |
| --- | --- | --- | --- | --- | --- |
| *APOE* genotypes | | | | | |
| ε2/ε2 | 0 | 0 | 0 | | 0 |
| ε2/ε3 | 16 (11.3%) | 3 (18.8%) | 14 (11.0%) | | 33 (11.6%) |
| ε2/ε4 | 6 (4.2%) | 0 | 6 (4.7%) | | 12 (4.2%) |
| ε3/ε3 | 88 (62.0%) | 11 (68.8%) | 78 (61.4%) | | 177 (62.1%) |
| ε3/ε4 | 29 (20.4%) | 2 (12.5%) | 26 (20.5%) | | 57 (20.0%) |
| ε4/ε4 | 3 (2.1%) | 0 | 3 (2.4%) | | 6 (2.1%) |
| *APOE* alleles |  | |  | |  |
| ε2 | 22 (7.8%) | 3 (9.4%) | 20 (7.9%) | | 45 (7.9%) |
| ε3 | 221 (77.8%) | 27 (84.4%) | 196 (77.2%) | | 444 (77.9%) |
| ε4 | 41 (14.4%) | 2 (6.3%) | 38 (15.0%) | | 81 (14.2%) |
| Total | 284 | 32 | 254 | | 570 |
| PolyT genotypes | | | | | |
| S-S | 32 (22.5%) | 4 (25.0%) | 22 (17.3%) | 58 (20.4%) | |
| S-L | 25 (17.6%) | 2 (12.5%) | 17 (13.4%) | 44 (15.4%) | |
| S-VL | 53 (37.3%) | 8 (50.0%) | 42 (33.1%) | 103 (36.1%) | |
| L-L | 10 (7.0%) | 1 (6.3%) | 14 (11.0%) | 25 (8.8%) | |
| L-VL | 5 (3.5%) | 0 | 9 (7.1%) | 14 (4.9%) | |
| VL-VL | 17 (12.0%) | 1 (6.3%) | 23 (18.1%) | 41 (14.4%) | |
| PolyT alleles* | | | | | |
| S | 142 (50.0%) | 18 (56.3%) | 103 (40.6%) | 245 (46.1%) | |
| L | 50 (17.6%) | 4 (12.5%) | 54 (21.3%) | 104 (18.9%) | |
| VL | 92 (32.4%) | 10 (31.3%) | 97 (38.2%) | 189 (34.9%) | |
| Total | 284 | 32 | 254 | 570 | |

*S: short polyT repeat (≤ 19 bp); L: long polyT repeat (20-29 bp); VL: very long polyT repeat (≥ 30 bp) in *TOMM40* gene.

**Supplementary Table 2. Frequencies of *TOMM40* polyT repeat genotypes in sIBM patients and controls stratified by *APOE* genotypes.**

|  | *APOE* ε2/ε3* | | | *APOE* ε2/ε4* | | | *APOE* ε3/ε3* | | | *APOE* ε3/ε4* | | | *APOE* ε4/ε4* | | |
| --- | --- | --- | --- | --- | --- | --- | --- | --- | --- | --- | --- | --- | --- | --- | --- |
| *TOMM40* polyT | **sIBM** | **Controls** | **Total** | **sIBM** | **Controls** | **Total** | **sIBM** | **Controls** | **Total** | **sIBM** | **Controls** | **Total** | **sIBM** | **Controls** | **Total** |
| S-S | 5  (26.3%) | 1  (7.1%) | 6  (18.2%) | 0 | 0 | 0 | 27  (27.3%) | 19  (24.4%) | 46  (26.0%) | 3  (9.7%) | 2  (7.7%) | 5  (8.8%) | 1  (33.3%) | 0 | 1  (16.7%) |
| S-L | 1  (5.3%) | 2  (14.3%) | 3  (9.1%) | 3  (50.0%) | 3  (50.0%) | 6  (50.0%) | 10  (10.1%) | 4  (5.1%) | 14  (7.9%) | 13  (41.9%) | 8  (30.8%) | 21  (36.8%) | 0 | 0 | 0 |
| S-VL | 7  (36.8%) | 7  (50.0%) | 14  (42.4%) | 0 | 0 | 0 | 48  (48.5%) | 34  (43.6%) | 82  (46.3%) | 6  (19.4%) | 1  (3.9%) | 7  (12.3%) | 0 | 0 | 0 |
| L-L | 0 | 0 | 0 | 1  (16.7%) | 2  (33.3%) | 3  (25.0%) | 1  (1.0%) | 3  (3.9%) | 4  (2.3%) | 7  (22.6%) | 6  (23.1%) | 13  (22.8%) | 2  (66.7%) | 3  (100%) | 5  (83.3%) |
| L-VL | 0 | 0 | 0 | 2  (33.3%) | 1  (16.7%) | 3  (25.0%) | 1  (1.0%) | 0 | 1  (0.6%) | 2  (6.5%) | 8  (30.8%) | 10  (17.5%) | 0 | 0 | 0 |
| VL-VL | 6  (31.6%) | 4  (28.6%) | 10 (30.3%) | 0 | 0 | 0 | 12  (12.1%) | 18 (23.1%) | 30  (17.0%) | 0 | 1  (3.9%) | 1  (1.8%) | 0 | 0 | 0 |
| Total | **19** | 14 | 33 | **6** | 6 | 12 | **99** | 78 | 177 | **31** | 26 | 57 | **3** | 3 | 6 |
| **p*>0.05 for the comparison between patients and controls within each *APOE* genotype (Fisher’s exact test). | | | | | | | | | | | | | | | |

**Supplementary Figure 1. Distribution of *TOMM40* polyT repeat lengths in the sIBM patient cohort (N=158) and in the control group (N=127).**
